# Changes in Physician Telemedicine Use during COVID-19: Effects of Practice Setting, Demographics, Training, and Organizational Policies

**DOI:** 10.3390/ijerph18199963

**Published:** 2021-09-22

**Authors:** Bradford S. Pierce, Paul B. Perrin, Alan W. Dow, Natalie D. Dautovich, Bruce D. Rybarczyk, Vimal K. Mishra

**Affiliations:** 1 Department of Psychology, Virginia Commonwealth University, Richmond, VA 23284, USA; piercebs@vcu.edu (B.S.P.); ndautovich@vcu.edu (N.D.D.); bdrybarczyk@vcu.edu (B.D.R.); 2 Department of Physical Medicine and Rehabilitation, Virginia Commonwealth University, Richmond, VA 23284, USA; 3 Division of Hospital Medicine, Virginia Commonwealth University, Richmond, VA 23284, USA; alan.dow@vcuhealth.org; 4 Department of Health Sciences for Interprofessional Education & Collaborative Care, Virginia Commowealth University, Richmond, VA 23284, USA; 5 Department of Medicine and Health Administration, Virginia Commonwealth University, Richmond, VA 23284, USA; vimal.mishra@vcuhealth.org; 6 Office of Telemedicine, Virginia Commonwealth University, Richmond, VA 23284, USA

**Keywords:** telemedicine, telehealth, COVID-19, physician

## Abstract

Telemedicine use increased during the COVID-19 pandemic, but uptake was uneven and future use is uncertain. This study, then, examined the ability of personal and environmental variables to predict telemedicine adoption during the COVID-19 pandemic. A total of 230 physicians practicing in the U.S. completed questions concerning personal and environmental characteristics, as well as telemedicine use at three time points: pre-pandemic, during the pandemic, and anticipated future use. Associations between use and characteristics were determined to identify factors important for telemedicine use. Physicians reported that telemedicine accounted for 3.72% of clinical work prior to the pandemic, 46.03% during the pandemic, and predicted 25.44% after the pandemic ends. Physicians within hospitals reported less increase in telemedicine use during the pandemic than within group practice (*p* = 0.016) and less increase in use at hospitals compared to academic medical centers (*p* = 0.027) and group practice (*p* = 0.008). Greater telemedicine use was associated with more years in practice (*p* = 0.009), supportive organizational policies (*p* = 0.001), organizational encouragement (*p* = 0.003), expectations of greater patient volume (*p* = 0.003), and perceived higher quality of patient care (*p* = 0.032). Characteristics such as gender, number of physicians, and level of telemedicine training were not significant predictors. Organizations interested in supporting physicians to adopt telemedicine should encourage its use and create policies supporting its use. More senior physicians had a greater degree of telemedicine uptake, while training programs did not predict use, suggesting that efforts to develop telemedicine competency in younger physicians may be ineffective and should be re-examined.

## 1. Introduction

Despite telemedicine’s history of connecting physicians with distant patients [1,2] few physicians practiced regularly with telemedicine before the COVID-19 pandemic [3]. The terms telehealth and telemedicine are often used interchangeably, but the U.S. Health and Services Administration describes *telehealth* as a broad range of technologies to provide healthcare, health-related education, and administration at a distance [4]. The Centers for Medicaid and Medicare Services defines *telemedicine* as “two-way, real-time interactive communication between the patient, and the physician or practitioner at the distant site” [5].

On an individual level, physicians evaluating telemedicine’s place within their practice must consider several factors. These concerns include avoiding unlicensed interjurisdictional practice [6], complying with telemedicine ethical guidelines and competencies outlined by the American Medical Association (AMA) [7], telemedicine-specific aspects of the Ryan Haight Act [8], and Medicare limits to telemedicine reimbursement [9]. Additionally, physicians cite lack of training, equipment costs, and increased liability among their most important concerns [10]. 

Amidst this backdrop, on 20 January 2020, the Centers for Disease Control and Prevention (CDC) confirmed that a patient within the U.S. had tested positive for COVID-19 [11]. Less than a month later, policy-level changes occurred once the World Health Organization (WHO) characterized COVID-19 as a pandemic [12]. For example, the CDC and the American College of Surgeons recommended healthcare clinics and facilities postpone elective procedures and routine visits [13,14]. Organizations such as the Veterans Health Administration (VHA), Mayo Clinic, and Johns Hopkins Health System greatly expanded their use of telemedicine [15,16,17]. Agencies within the U.S. relaxed established policies including the ‘in-person’ requirement in the Ryan Haight Act [18] while Medicare and Medicaid began reimbursing telemedicine visits with patients across the country, including within patients’ homes, and at the same rates as in-person visits [19]. 

A survey conducted by the *COVID-19 Healthcare Coalition Telehealth Impact Study Work Group* in November 2020, found that telemedicine use increased dramatically during the pandemic. This survey reported platforms and technologies and types of services conducted with telemedicine as well as perceived benefits and barriers to long-term use of telemedicine and types of patients best suited to it in the future. Chronic disease care and prevention services were anticipated by a large margin to be the type of service most likely to be provided via telemedicine. The study reported differences in perceptions between urban, suburban, and rural physicians but did not provide predictive modeling of these differences or examine other factors contributing to use of telemedicine [20]. 

The current study had multiple aims. The first was to examine whether physicians’ use of telemedicine changed from before the COVID-19 pandemic to during the pandemic, as well as whether physicians projected additional changes in their telemedicine use after the pandemic ended. The second aim was to examine the ability of personal and environmental variables to predict telemedicine adoption during the COVID-19 pandemic.

## 2. Method

### 2.1. Participants

This study was reviewed by the Virginia Commonwealth University Independent Review Board (IRB) to ensure it was conducted ethically and in compliance with all federal, state, and local regulations concerning research involving human participants. Recruitment used email addresses from directories of professional organizations, hospital and health clinic websites, and professional newsgroups and social media groups. Eligibility requirements were that participants were: (a) licensed to practice as a physician in the USA, (b) age 18 or older, and (c) currently practicing (seeing patients) as a physician in the USA. Data were collected from 12 May 2020, to 25 July 2020. Initial and follow-up email invitations were sent to 850 individuals and posted to online groups of physicians, with 46 emails returning as ‘undeliverable.’ A total of 315 people (representing 39.2 percent of received invitations) opened the survey, of which 21 left after viewing the information sheet. Participant data were reviewed to determine eligibility and missingness, resulting in a sample size of 230 licensed, currently practicing physicians (Table 1).

### 2.2. Measures

Participants provided demographic and practice-related information. Additionally, participants were asked to provide responses regarding their telemedicine use, training, and organizational policies after 20 January 2020, when the first COVID-19 case was confirmed in the USA. “Telemedicine” was defined to participants as “the use of real-time audio (e.g., telephone) and/or video conferencing technology to provide healthcare services.”

The researcher-generated telemedicine question used was: “What percentage of your patient treatment is provided using telemedicine?” with answers ranging from 0 to 100%. Participants were instructed to respond three times with regard to the following prompts: (a) “Before the COVID-19 pandemic began in the USA on 20 January 2020”; (b) “During the COVID-19 pandemic in the USA”; and (c) “Your anticipated perspective or behaviors after the COVID-19 pandemic ends in the USA”. The amount of change for the current primary analyses was determined by subtracting participants’ responses about their telemedicine use before the pandemic from their answers during the pandemic.

### 2.3. Facilitators of Telemedicine Use

A set of 10 items tapping potential facilitators of telemedicine use was developed through consultation with two physicians with expertise overseeing the rollout of telemedicine services in a large healthcare system. These items addressed issues concerning quantity and quality of patient care, training, policy support, reimbursement, infrastructure support, and level of organizational support. Participants responded on a 7-point Likert-type scale ranging from 1 (“strongly disagree”) to 7 (“strongly agree”) differentially with regard to the same three time points specified in the rest of the survey. In the current sample, these items demonstrated good internal reliability for ratings before (α = 0.87) and during (α = 0.84) the pandemic (α = 0.85). As before, the amount of change in these variables for the primary analyses was determined by subtracting participants’ responses regarding before the pandemic from their answers regarding during the pandemic.

### 2.4. Procedure

Potential participants were sent an initial recruitment email, and a follow-up email one week later, inviting them to complete a 10-min survey that would “help inform treatment approaches used during the pandemic, as well as public healthcare policy.” To avoid biasing enrollment based on preconceived notions of telemedicine, no reference to telehealth or telemedicine was made in the recruitment email or informed consent document.

### 2.5. Data Analyses

All analyses were conducted using SPSS Version 27.0 (IBM, Armonk, NY, USA) [21]. Significance was established at an alpha level of 0.05, two-tailed. Assumption violations were reported and analyses were adjusted by using commonly used conservative approaches. A repeated-measures analysis of variance (ANOVA) examining the effects of time on the percentage of clinical work performed via telemedicine was conducted. A series of one-way ANOVAs then compared the percentage of telemedicine use and change in telemedicine use among primary practice settings. These latter ANOVAs included only participants who worked in primary treatment settings selected by 30 or more participants, and participants who chose the “other” designation were excluded.

Next, two multiple regression analyses were conducted to examine the effects of (a) participant demographics, change in workplace telemedicine policy, change in telemedicine training, and geographic area, as well as (b) a set of potential facilitators for telemedicine use on change in telemedicine adoption. In both regressions, the primary variable of interest was the percentage of clinical work conducted via telemedicine during the pandemic minus the percentage conducted via telemedicine before the pandemic. The first model’s predictors included years of experience, gender (1 = woman, 0 = man), practice setting (1 = academic medical center, 0 = other), change in perceived organizational support via telemedicine policies, change in perceived levels of telemedicine training received, and the number of physicians within the practice setting. The second model’s predictors included pre–during pandemic change in physicians’ level of agreement from 1 (“strongly disagree”) to 7 (“strongly agree”), with ten statements representing potential facilitators of telemedicine use.

## 3. Results

### 3.1. Changes in Telemedicine Use over Time

**Figure 1:**
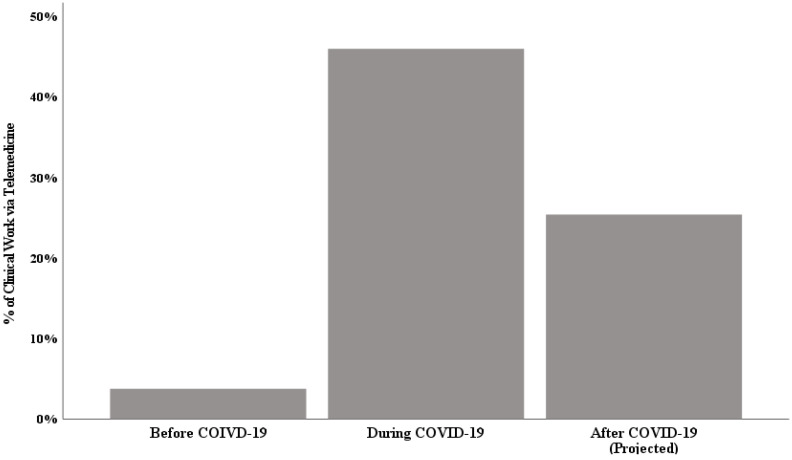
Estimated percentage of clinical work performed via telemedicine before, during, and after (projected) the COVID-19 pandemic.

The repeated-measures ANOVA indicated that differences in the percent of clinical work performed by telemedicine over time were statistically significant, *F*(1.47, 522.27) = 268.07, *p* < 0.001, partial η^2^ = 0.592. Before the COVID-19 pandemic, physicians performed 3.72% (*SD* = 13.70) of their clinical work with telemedicine, 46.03% (*SD* = 35.74) during the pandemic, and a projected 25.44% (*SD* = 24.64) after the pandemic (Figure 1). Relative to pre-pandemic use of telemedicine, these changes represented a more than 12-fold increase in telemedicine use during the pandemic, and a nearly 8-fold increase in anticipated use after the pandemic.

### 3.2. Primary Practice Setting

Results of one-way ANOVAs comparing the percentage of telemedicine use and change in telemedicine use among physicians in primary practice settings with 30 or more participants appear in Table 2. In the analysis of telemedicine use before the COVID-19 pandemic, the assumption of homogeneity of variance was violated, so *F* was calculated using a more conservative approach proposed by Welch [22]. The differences in use among the three settings were not significantly different prior to the pandemic, *F* (2, 64.14) = 0.93, *p* = 0.399. There were significant differences, however, during the pandemic, *F* (2, 182) = 4.50, *p* = 0.012. Follow-up comparisons indicated that physicians within a group practice reported higher levels of telemedicine use than those in hospitals (*p* = 0.016). There were also significant differences in levels of change in telemedicine use percentage prior to the pandemic to during the pandemic between groups (*p* =0.004). Physicians within hospitals reported a smaller percentage increase in telemedicine use than those in academic medical centers (*p* = 0.027) and group practice (*p* = 0.008). Physicians in hospitals and academic medical centers each reported a more than 29-fold increase in telemedicine use during the pandemic compared to pre-pandemic levels, while those within group practice experienced a more than 12-fold increase in telemedicine use (Table 2). While physicians in each of these settings anticipated higher levels once the pandemic ended compared to pre-pandemic levels, no significant difference was detected between them (*p* = 0.065).

### 3.3. Demographics, Training, and Organizational Policies

Demographic, training, and organizational policy variables used as predictors within the first multiple regression model were analyzed to determine the nature of any bivariate relationships among each other and with change in telemedicine use, as well as to verify that none of them correlated with each other too highly to the point of multicollinearity. Results appear in Table 3.

The multiple regression model provided a statistically significant prediction of change in telemedicine use during the COVID-19 pandemic relative to before (*p* < 0.001). When controlling for the other predictors, physicians with more supportive organizational telemedicine policies had a larger increase in telemedicine use (*p* = 0.001). Physicians with more years in practice also reported a larger increase in telemedicine use than younger physicians (*p* = 0.009). Although training was significantly correlated with increased use (*p* = 0.002), its unique influence was not significant when these other predictors were accounted for. No other predictors exerted a unique effect on increase in telemedicine use (Table 4).

### 3.4. Facilitators of Telemedicine Use

The second multiple regression model provided a statistically significant prediction of change in telemedicine use during the COVID-19 pandemic relative to before (*p* < 0.001). When controlling for the other predictors, an increase in perceived patient volume was associated with increased telemedicine use (*p* = 0.003). An increase in perceived telemedicine-care quality rating correlated with a greater increase in telemedicine use (*p* = 0.032). Additionally, an increase in perceived organizational encouragement resulted in a greater increase in telemedicine use (*p* = 0.003). No other predictors exerted a unique effect on increase in telemedicine use (Table 5).

## 4. Discussion

This study described physicians’ adoption of telemedicine during the COVID-19 pandemic and examined key issues that influenced physicians’ adoption of telemedicine. The findings documented the extraordinary shift in healthcare delivery among physicians in such a short time period. Compared to prior to the pandemic, physicians were far more likely to use telemedicine during the pandemic and they anticipated a higher proportion of patient care would occur via telemedicine once the pandemic ends. The results also indicated that physicians were more likely to use telemedicine when they had more years of experience, when working in organizations with more supportive telemedicine policies and encouragement to use it, and when they believed it would result in greater patient volume and improved patient care quality.

Physicians reported that telemedicine accounted for 3.72% of their clinical work with patients before the pandemic, 46.03% during the pandemic, and projected it would account for 25.44% their work with patients after the pandemic. Physicians’ level of use prior to the pandemic was far less than that found in the AMA’s 2016 *Physician Practice Benchmark Survey*, within which 15.4% of physicians reported working in settings that used telemedicine [3].

The increase in telemedicine use during the pandemic conforms to expectations based on several factors. First was the need for people to physically distance from one another to reduce the risk of transmitting the COVID-19 virus, especially among people with preexisting medical conditions. Furthermore, some federal regulations and Medicare reimbursement policies were relaxed within the USA allowing physicians to practice telemedicine in situations that were previously prohibited or for which they could not be reimbursed [18,19]. The need to avoid unnecessary in-person contact during the pandemic and the suspension of long-standing barriers meant that telemedicine was, at least during the pandemic, a viable alternative for some patient care. The anticipated decrease in telemedicine use after the pandemic may be partly attributable to physicians’ predicting reinstatement of some policies that were restricting the use of telemedicine prior to the pandemic. For example, CMS indicated they were considering eliminating reimbursement for some telemedicine services at the end of the calendar year once the pandemic-related emergency ends [23]. 

Contrary to what was anticipated, a greater number of years in practice was also associated with a greater percentage of telemedicine use. In a pre-pandemic survey distributed by the American Well [24], early career physicians reported they were less likely to use telehealth. The authors hypothesized that these physicians were still focused on learning more basic aspects of their craft, so they might not be as receptive to the additional complexity that comes with telehealth use.

Although there were no differences in telemedicine use among physicians working within hospitals, academic medical centers, and group practice prior to, or after the COVID-19 pandemic, there were differences observed during the pandemic. Those within group practice reported a higher percentage of telemedicine use than those within hospital settings. This was surprising considering a 2016 report to Congress estimating that about 40% to 50% of hospitals used some form of telemedicine [25]. Group practice settings often have a higher proportion of patients receiving outpatient treatment, while a larger percentage of patients receiving treatment from hospitals are located on site. With many patients already within the facility, telemedicine use may be less frequently needed.

Results of multiple regression analyses indicated that when physicians perceived an increase in organizational encouragement and supportive policies regarding telemedicine within their practice, they were more likely to use it. This aligns with previous studies indicating that supportive organizational policies share a positive relationship with telemedicine and telepsychology adoption [26,27,28]. Organizational policies communicate what is expected and what decisions the organization will support if issues arise.

Lastly, telemedicine use during the pandemic was higher when physicians perceived it would facilitate treating more patients while also improving care quality. This suggests physicians adopted a results-driven and patient-centered approach as they evaluated telemedicine use. These results dovetail with previous research indicating that physicians are strongly influenced by the utility of a technology [29]. 

### 4.1. Implications

The results of this study have a number of implications. First, the findings reveal some of the key considerations for physicians as they evaluated whether telemedicine was appropriate within their own practice during the pandemic. Among these were telemedicine’s ability to help physicians treat more patients, the quality of care they could deliver with it, aspects of their practice setting, and the strength of telemedicine policies in place. These topics can guide groups interested in supporting physicians in using telemedicine when it is appropriate to do so.

Organizations promoting telemedicine use should craft and effectively communicate policies concerning telemedicine use. The results highlight the importance of training and gaining support for telemedicine from more experienced physicians within a group. Senior physicians may be more likely to incorporate telemedicine into their practice. Furthermore, it is important to provide information demonstrating telemedicine’s effectiveness. Larger professional organizations could promote efficacy studies and share information about how physicians in various settings or practices incorporate telemedicine into their practice.

Finally, the results also contribute to research regarding physicians’ flexibility and their motivations as they adjust patient care within unusual local and global circumstances. Healthcare professionals endured a considerable amount of stress during the COVID-19 pandemic as they adjusted to new situations in their work. In addition to the anxiety faced by everyone within the greater community, care providers were concerned about limited protective gear, confronted difficult moral dilemmas when resources and personnel were scarce, and worried about the greater risk of exposure for themselves and their families when they returned home [30]. Physicians are called upon to adapt treatment to unexpected situations including disaster areas, zones of conflict, and remote locations [31]. They do not do this in a vacuum. Government institutions within the USA made shifts in several policies to allow for greater flexibility in how and where telemedicine was conducted. It is unlikely that rapid deployment of telemedicine would have been possible without these policy changes. Physicians anticipated approximately 25% of treatment will occur via telemedicine after the pandemic. They saw a place for it within their practice. Considering that many of the government policy changes have been described as temporary, these results may be used to support retaining many of these changes.

### 4.2. Limitations and Future Directions

This study has several limitations that should be acknowledged, as well as potential directions for future research. First, is that internet-based surveys can lead to bias within the sample since the investigators had very little control over who received their invitations and decided to participate [32,33]. For example, while 63.9% of participants within this sample identified as women, the Association of American Medical Colleges reports that only 36.3% of physicians practicing within the USA in 2019 identified as women [34]. This difference indicates the study’s sample does not reflect their roster of physicians. Availability bias is particularly important to consider in this case since many individuals working within busier healthcare settings were under considerable stress. It is very likely, and appropriate, that physicians who were coping with patient surges and limited resources were unavailable to participate in this study.

One aspect of the study deserving consideration is that physicians were asked to make predictions concerning telemedicine after the pandemic ended. These predictions do not equate to a ‘lived experience’ by the participants, and as such, their responses should be viewed as speculative rather than reflecting reality. A follow-up study conducted once the pandemic ends would more accurately document physician experiences.

Another issue was that the study only focused on physicians’ experiences and perceptions of telemedicine. In 2019, nurse practitioners (NP), and physician assistants (PA) represented approximately 336,000, of those practicing medicine within the USA [35,36]. NP and PA positions were originally created to address the physician shortages within areas such as rural regions and inner-city settings [37]. Considering their important role concerning patient care and the populations that they treat, their beliefs, expectations, and experiences are important for gathering a more comprehensive understanding of telemedicine’s place within patient care. Future studies could broaden inclusion criteria to allow NPs and PAs to add their perspective.

## 5. Conclusions

Telemedicine has the potential to help physicians reach patients who are inhibited by circumstances from receiving in-person medical treatment. Prior to the COVD-19 pandemic, telemedicine was used very little by USA physicians. Use increased dramatically during the pandemic, and physicians predicted they would rely on it much more after the pandemic ends than they had prior to the pandemic. These results can help guide government institutions, healthcare organizations, and other physicians as they persuade physicians to consider telemedicine’s place. Doing so may help them treat people limited by circumstances from receiving in-person treatment.

## Figures and Tables

**Table 1:** Summary of Participant Characteristics.

Characteristics		
**Age**, *M*, *SD*	46.21	10.16
**Years in Practice**, *M*, *SD*	18.27	10.00
**Gender**, *n*, %		
Woman	147	63.9
Man	83	36.1
**Race/Ethnicity**, *n*, %		
White/European-American (NH/NL)	172	74.8
Asian/Asian-American (NH/NL)	32	13.9
Latinx/Hispanic	9	3.9
Multiracial/Multiethnic	8	3.5
Black/African-American (NH/NL)	6	2.6
Other	2	0.9
American Indian/Alaska Native/Native American (NH/NL)	1	0.4
**Primary Practice Setting**, *n*, %		
Academic Medical Center	94	40.9
Hospital	58	25.2
Group Practice	33	14.3
Other	16	7.8
School/University	8	3.5
Outpatient Treatment Facility	7	3.0
Veterans Affairs Medical Center	7	3.0
Individual Practice	6	2.6
Health Maintenance Organization	1	0.4
**Practice Location**, *n*, %		
Urban	154	67.0
Suburban	59	25.7
Rural	17	7.4
**Number of Physicians in Practice**, *n*, %		
1	8	3.5
2–5	47	20.4
6–10	53	23.0
11–20	31	13.5
21–50	21	9.1
50+	70	30.4

Note. NH/NL = Non-Hispanic/Non-Latinx.

**Table 2:** Percentage use of telemedicine by primary practice setting.

	% Use before COVID-19	% Use during COVID-19	Change in % Use during COVID-19	Projected % Use after COVID-19
Omnibus ANOVA *p*-value	0.399	0.012	0.004	0.065
Variable				
Hospital	3.71%	_a_ 31.72%	_ab_ 28.02%	17.29%
Academic Medical Center	1.52%	44.75%	_a_ 43.22%	25.52%
Group Practice	4.37%	_a_ 53.42%	_b_ 51.09%	25.91%

Note. Percentages within a column sharing the same subscript (were significantly different at *p* < 0.05 after Bonferroni corrections.

**Table 3:** Correlation matrix of continuous demographic variables.

Variables	1	2	3	4	5	6
1. Change in Telemedicine Use	-					
2. Years in Practice	0.117 *	-				
3. Identifies as a Man	−0.119	0.194 **	-			
4. Academic Medical Center Setting	0.035	0.028	0.185 **	-		
5. Physicians in Setting	0.060	−0.047	0.214 **	0.332 **	-	
6. Supportive Policies	0.300 **	−0.094	−0.168 **	0.000	−0.039	-
7. Training	0.187 *	−0.005	−0.004	−0.002	0.049	0.344 **

*Note*: * = *p* < 0.05; ** *p* < 0.01.

**Table 4:** Multiple regression of demographic, training, and organizational policy predictors.

Variable	B	S.E.	β	Sig.
Years in Practice	0.591	0.225	0.169	0.009
Identifies as a Man	−7.992	4.861	−0.110	0.102
Academic Medical Center Setting	4.706	4.796	0.066	0.328
Number of Physicians in Setting	−0.961	1.441	−0.045	0.506
Supportive Telemedicine Policies	4.622	1.206	0.261	0.001
Sufficient Telemedicine Training	1.973	1.323	0.100	0.137
Constant	22.513	7.867		0.005

**Table 5:** Multiple regression of facilitators of telemedicine use.

Variable	B	S.E.	β	Sig.
Patient Volume	3.663	1.216	0.196	0.003
Training Received	−0.078	1.638	−0.004	0.962
Available Equipment	1.204	1.707	0.059	0.481
Care Quality	3.784	1.752	0.165	0.032
Reimbursement	0.709	1.480	0.036	0.632
Supportive Policies	−0.705	1.710	−0.040	0.681
Supportive Regulations	1.266	1.404	0.073	0.368
Training Offered	−2.472	1.968	−0.111	0.210
Technical Support	−1.193	1.826	−0.056	0.514
Encouraging Org	4.773	1.609	0.277	0.003
Constant	17.679	14.380		0.000

## Data Availability

The data presented in this study are available on reasonable request from the corresponding author. The data are not publicly available due to privacy or ethical considerations.
